# Parental political engagement and self‐efficacy: An exploratory study

**DOI:** 10.1002/ajcp.12761

**Published:** 2024-07-01

**Authors:** Christina Randolph, Robert Needlman, Ellen Hutchinson

**Affiliations:** ^1^ School of Medicine Case Western Reserve University Cleveland Ohio USA; ^2^ The MetroHealth System Cleveland Ohio USA; ^3^ University Hospitals‐Rainbow Babies and Children's Hospital Cleveland Ohio

**Keywords:** political engagement, parenting, self‐efficacy

## Abstract

Political engagement (PE) is associated with a sense of empowerment. PE by parents affects children's lives. This study explored parents' attitudes about inclusion of political engagement discussion in well‐child care. Because voting is an expression of empowerment, we hypothesized that voting would be related to higher parenting self‐efficacy. We administered a brief questionnaire to a convenience sample of parents/guardians at well‐child visits at an urban hospital. The questionnaire included 10 Likert scale questions touching on parenting efficacy; beliefs about political engagement; voting behaviors; and demographics. Analysis included descriptive statistics, correlations, and factor analysis. Among the 70 respondents 84% were mothers; 55% self‐identified as low income; 62% self‐identified as a “person of color.” Voting in the 2020 national election was reported by 37%. Most parents (54%) said they would feel comfortable discussing voting with a doctor. Factor analysis identified two main factors: self‐efficacy and engagement. Engagement, but not self‐efficacy, was related to voting behavior. Contrary to our hypothesis, PE did not appear related to parenting self‐efficacy. Notably, more than half of parents felt comfortable discussing PE with their child's doctor. Given the critical role of parents in shaping the world their children inhabit, further exploration could prove to be of value.

## Highlights

- In this study voting behavior was related to political engagement beliefs, but not self‐efficacy.
- In this study most parents were open to discussing voting with their child's health care provider.
- Further exploration is warranted in the potential relationship between parental Political engagement and self‐efficacy.

## INTRODUCTION

Political engagement (PE) involves the broad range of activities that shape the political process (Khasnabis et al., 2010). PE impacts the leadership and decision making that shapes our world. PE comes in many forms including campaigning, donating, attending political events, lobbying, and voting. At least two thirds of Americans participate in political engagement activities (Oliphant, 2018).

Pediatricians have historically been involved in political engagement through advocacy, which is important because politics affects children. For example, policies on supplemental nutrition access, health insurance coverage, and reproductive health affect youth. Parents are also important in influencing policy. Through parent organizing, the Parent Voices group was able to achieve legislative change in child care funding (“Who We Are,” 2023). Additionally, children of politically engaged parents are more likely to be politically engaged themselves when they grow up (Gidengil et al., 2016). Thus, PE by parents is important because their actions can both affect current policies as well as have downstream affects by modeling PE for their children.

Of the various forms of PE, voting is the quintessential and is the most readily measured. Rates of voter turnout vary within society. Voting is less common among people with lower socioeconomic status, less education, people of color, and young people (“Who Votes, Who Doesn't, and Why,”, 2006). Barriers include apathy, lack of knowledge of candidates, lack of trust, variability in campaign outreach, social isolation and difficulty getting to a polling place (“Who Votes, Who Doesn't, and Why,”, 2006). Some of these factors may be related to parents' PE.

The health care setting is a place that can help encourage PE by parents. In a federally qualified health center in New York, waiting room volunteers were able to register 89% of eligible but previously unregistered voters (Liggett et al., 2014). Another project, the Vot‐ER project provided kiosks and QR codes on posters to help both staff and visitors register to vote, a project that continues to be active at over 100 sites (Peeples, 2020). Several national bodies endorse engaging patients about voting, including the AMA and AAP (Chomilo et al., 2021; Why It's OK for Doctors to Ask Their Patients about Voting, 2021). Thus, there is a precedent for health organizations promoting voting, and doctors can play a pivotal role.

Given health systems' potential influence on parental PE, and the influence of parental PE on society, we wanted to understand parents' thoughts about political engagement and how they might feel about discussing voting with their child's clinician. Discussing voting with a doctor may mobilize more parents to be politically active. Some parents may be open to this discussion. However, other parents might find such topics to be intrusive. The first aim of this study was to explore how parents in an urban pediatric clinic might feel about doctors initiating a discussion on voting behavior during health supervision visits.

The second aim was to better understand factors that might increase or decrease parental PE. Self‐efficacy is related to locus of control (LOC), and in the context of parenting, parental locus of control (PLOC). LOC and political participation are related (Majete, 1987; Rosen & Salling, 1971). Similarly, certain life stressors may influence PE (Hassell & Settle, 2017; Hobbs et al., 2014; Pacheco & Plutzer, 2007; Sandell & Plutzer, 2005). However, the literature is silent on attitudes around parenting stress and its relation to parental PE. We hypothesized that voting (i.e., empowerment in the political sphere) would be associated with higher parenting self‐efficacy (i.e. empowerment within the parenting sphere), and lower parenting stress.

## METHODS

### Questionnaire design

PLOC surveys were reviewed in the literature. The current survey was a pilot study, where questions specific to parenting stress, PLOC and voting behavior were crafted based on the constructs and themes of PLOC. To maximize the completion rate in our convenience sample of parents, we designed the survey to be very short.

These considerations led to the development of the Parents' Thoughts About Voting Questionnaire (PTVQ). The survey included ten Likert scale questions related to parenting self‐efficacy and political engagement as well as parental demographic information (Appendix A). The questionnaire includes a measure of LOC (question 1), a measure of PLOC (question 2) and a measure of parenting stress (question 3). Questions 4 and 6 included parent's general views on voting. Questions 7 and 8 discussed parents’ thoughts about discussing voting with a doctor. Questions 5, 9 and 10 also covered parents’ thoughts about voting, particularly in regard to how it could affect children. Demographics included parenting role, self‐reported income group and identity as “person of color” versus “white,” and a marker of age. It also included questions of whether the participant voted in the 2018 congressional election and 2020 major election. Participants were able to circle options of why they did not vote, or write in other reasons.

### Participant selection/recruitment

Study participants were selected via convenience sampling. Participants were parents/guardians of children to the ambulatory pediatric department in an urban safety‐net hospital. “Parent/guardian” included mothers, fathers, grandparents, step‐parents, and foster parents. Nonparent/guardians were excluded from the study. Parents under the age of 18 were also excluded. The survey was collected over a 3‐month time period based on pediatric clinics open during researchers' availability.

### Survey dissemination/data collection

Researchers introduced the study to parents at the pediatric visit as they were awaiting care by a provider. Participation in the study was voluntary. The researcher gave the parent a paper survey inside of an envelope and asked the parent to complete the survey. Informed consent without signature via a nonreturn cover memo was provided to parents with the survey. The envelope with the survey was collected by either the researchers or clinical staff and placed in a survey collection bin by the end of the visit. No patient identifiers were listed on the survey. The number of those declining to participate was not tracked. Only the number of collected surveys were tracked.

### Ethics/IRB

This study was approved as exempt status by the MetroHealth IRB (IRB20‐00796). Parents had the opportunity to ask questions and verbalized consent to participate in the study.

### Data analysis

Data were entered into Excel and then imported to SPSS for analysis. Parental age was calculated based on the parent's written year and month of birth, compared to the date the survey was completed. When the date was not provided on the survey, the date of the start of survey collection was used to estimate parental age. In SPSS, descriptive statistics of Likert scale questions and demographic variables were performed. Likert scale questions were recategorized into yes/no questions using a scale of 1–5 = no and 6–10 = yes and compared using Pearson correlation to identify associations between individual variables. Exploratory factor analysis was performed to identify subscales within the pilot screener. For the exploratory factor analysis, a principal components extraction method with Varimax with Kaiser Normalization was performed.

## RESULTS

### Demographic statistics

Seventy individuals participated in this pilot study. Not all participants opted to answer every question. The demographics of the participants are outlined in Table 1. Of those who responded, 84% of participants were mothers. Fifty‐five percent of participants identified as low income versus middle income (45%). Sixty‐two percent identified as a person of color versus not a person of color (38%). Parent/guardian age ranged from 19 to 54, with an average and median of 31 years. Likert scale questions with averages reported are shown in Table 2. There were no statistically different responses in Likert scale answer choices based on income or race.

**Table 1:** Demographics of study participants.

Demographic variable	Count (Percent)
**Parenting role:**	
Mother	59 (84.3)
Father	5 (7.1)
Stepparent	2 (2.9)
Grandparent	2 (2.9)
No answer	2 (2.9)
**Self‐reported income:**	
Lower	32 (45.7)
Middle	26 (37)
No answer	12 (17.1)
**Person of color:**	
Yes	34 (48.6)
No	21 (30)
No answer	15 (21.4)
**Electoral participation:**	
*2018 election:*	
Yes	24 (34.3)
No	38 (54.3)
No answer	8 (11.4)
*2018 election:*	
Yes	26 (37.1)
No	39 (55.7)
No answer	5 (7.1)

**Table 2:** Likert scale questions and average responses.

Survey question	Average score	Number (*N*)
I feel I am in control of my own life.	8.8	70
I feel I am in control of what happens to my child.	8.7	70
I feel very stressed by my responsibilities as a parent.	4.4	69
Voting is an important part of citizenship.	7.7	69
Voting by parents can make a difference in their children's health.	7.4	68
My vote matters.	7.7	70
I would feel comfortable talking about voting with my child's doctor, as long as we didn't talk about the sides.	6.5	69
Pediatricians and family doctors should ask parents about voting.	3.9	69
If more parents voted, children would be better off.	6.3	69
I want my child to grow up to be an engaged citizen.	8.4	68

### Voter participation

A similar proportion of respondents reported participation in the major 2020 election (37%) compared to the 2018 midterm election (34%); 27% reported voting in both elections. Voting in 2018 was associated with feeling that voting was important (*p* = .00), that voting can make a difference in their children's health (*p* = .02), that voting matters (*p* = .02), and that children would be better off if more parents voted (*p* = .02). Likewise, voting in 2020 was associated with feeling that voting was important (*p* = .01), makes a difference (*p* = .02), that talking to a doctor about voting was ok (*p* = .04), and that children would be better off if parents voted (*p* = .02). Voting in both elections was also associated the belief that voting is important (*p* = .00), makes a difference (*p* = .02), matters (*p* = .03), makes kids better off (*p* = .01), that talking to a doctor about voting is ok (*p* = .02), and that they want their kids to be an engaged citizen (*p* = .02). Voting in 2018, 2020, or both elections was not related to feeling in control of one's life, their child's life, or feeling stressed as a parent.

### Discussing voting with a doctor

Over half of parents (54%) felt comfortable discussing voting with a doctor, while nearly a quarter (24%) of parents felt doctors “should” include questions about voting in the visit. Independent samples T‐test showed that the belief that doctors should ask about voting was statistically significantly associated with feeling that voting is important (0.01), that voting matters (0.00), and that children are better off if more parents voted (0.00). Likewise, comfort in talking to their child's doctor was associated with less parenting stress (0.02), feeling that voting is important (0.02), feeling that voting matters (0.02), feeling that children are better off if parents vote (0.00), and wanting their child to grow up to be an engaged citizen (0.02).

### Pearson's correlation studies

The LOC measure (question 1) was moderately negatively correlated with self‐reported parenting stress (question 3) (*r* = −.32, *n* = 69, *p* = .01), showing that a greater feeling of control of one's life was related to less reported parenting stress. Apart from weaker associations related to asking about voting, generally most responses among questions 4–10 showed moderate associations between one another, such as the perception that voting is important was directly correlated with the belief that voting makes a difference (*r* = .62, *n* = 67, *p* = .00) and that voting matters (*r* = .66, *n* = 69, *p* = .00). Additionally, the perception that voting makes a difference was directly correlated with the perception that children would be better off if more parents voted (*r* = .63, *n* = 67, *p* = .00). Pearson correlation studies are displayed in Table 3.

**Table 3:** Pearson correlations among likert‐scale questions:.

	Own control	Child control	Stress	Important	Difference	Matters	Talk Ok	Ask	Better Off	Engaged
**Own control**										
Pearson Correlation	1	0.197	−0.323	0.123	0.155	0.267	0.155	0.110	0.011	0.104
Sig. (two‐tailed)		0.103	0.007	0.315	0.206	0.025	0.204	0.366	0.929	0.398
**Child control**										
Pearson correlation		1	−0.071	0.166	0.173	0.185	0.092	0.269	0.157	0.132
Sig. (two‐tailed)			0.560	0.173	0.159	0.126	0.454	0.026	0.198	0.283
**Stress**										
Pearson correlation			1	0.236	0.232	0.088	0.224	0.013	0.226	0.066
Sig. (two‐tailed)				0.053	0.058	0.472	0.064	0.915	0.062	0.592
**Important**										
Pearson correlation				1	0.619	0.665	0.517	0.342	0.567	0.460
Sig. (two‐tailed)					0.000	0.000	0.000	0.000	0.000	0.000
**Difference**										
Pearson correlation					1	0.750	0.325	0.196	0.635	0.533
Sig. (two‐tailed)						0.000	0.007	0.112	0.000	0.000
**Matters**										
Pearson correlation						1	0.428	0.384	0.608	0.483
Sig. (two‐tailed)							0.000	0.001	0.000	0.000
**Talk Ok**										
Pearson correlation							1	0.362	0.492	0.400
Sig. (two‐tailed)								0.002	0.000	0.001
**Ask**										
Pearson correlation								1	0.510	0.286
Sig. (two‐tailed)									0.000	0.018
**Better Off**										
Pearson correlation									1	0.506
Sig. (two‐tailed)										0.000
**Engaged**										
Pearson correlation										1
Sig. (two‐tailed)										

### Factor structuring

Exploratory factor analysis was performed on the PTVQ data. Two eigenvalues greater than 1.0 were found (4.1 and 1.5), which accounted for 56.7% of the variance. Rotated component matrix showed loads of 0.550‐0.845 on the intended factor for factor 1, engagement, and loads of −0.768‐0.774 on the intended factor for factor 2, self‐efficacy. For factor 2, the loads were generally above 0.4, except for question 3 (“I feel very stressed by my responsibilities as a parent.”), which showed the hypothesized negative correlation with self‐efficacy. Factor loadings on their intended factors are displayed in Table 4.

**Table 4:** Loadings of PTVQ items on their intended factor as obtained by means of exploratory factor analysis (principal components, varimax rotated).

Survey item	Initial factor analysis (component matrix)	Final factor analysis (rotation matrix)
*Self‐efficacy*		
I feel I am in control of my own life.	0.76	0.77
I feel I am in control of what happens to my child.	0.48	0.50
I feel very stressed by my responsibilities as a parent.	−0.79	−0.77
*Engagement*		
Voting is an important part of citizenship.	0.84	0.85
Voting by parents can make a difference in their children's health.	0.80	0.80
My vote matters.	0.86	0.85
I would feel comfortable talking about voting with my child's doctor, as long as we didn't talk about the sides.	0.65	0.66
Pediatricians and family doctors should ask parents about voting.	0.57	0.55
If more parents voted, children would be better off.	0.82	0.83
I want my child to grow up to be an engaged citizen.	0.70	0.69

### Factor analysis

Engagement (Factor 1) was associated with voting in elections (both or neither elections *p* = .01; 2020 election *p* = .01; 2018 election *p* = .01). The self‐efficacy factor (Factor 2) was not associated with voting in elections. Income, race, and age were not related to self‐efficacy or engagement factors. Also, the study found no intercorrelation between engagement and self‐efficacy (95%CI [−0.206–0.263]). Relationships between factors 1 (engagement) and 2 (self‐efficacy), voting behavior, and demographic variables are displayed in Table 5.

**Table 5:** Relationship between factors, political participation and sample demographics.

	Factor 1 (Engagement)				Factor 2 (Self‐Efficacy			
	Mean (SD)	F (Sig)	T (df)	*p*	Mean (SD)	F (Sig)	T (df)	*P*
**Voted in 2018**	1.47 (0.52)	4.52 (0.038)	2.84 (60.0)	.01	0.29 (0.45)	2.53 (0.12)	0.64 (60)	.53
**Voted in 2020**	1.45 (0.55)	2.99 (0.09)	2.64 (63.0)	.01	0.06 (0.43)	6.89 (0.01)	0.14 (62.57)	.89
**Voted in both versus neither**	1.80 (0.58)	5.84 (0.02)	3.09 (49.0)	.01	0.11 (0.46)	5.44 (0.02)	0.21 (49)	.81
**Income**	0.30 (0.56)	10.12 (0.00)	0.53 (50.29)	.60	−0.26 (0.45)	0.45 (0.51)	−0.58 (56)	.57
**Person of color**	0.37 (0.65)	2.70 (0.11)	0.57 (53.0)	.58	0.30 (0.56)	10.11 (0.00)	0.53 (50.29)	.60
**Age**	−0.89 (0.54)	0.67 (0.42)	1.63 (63.0)	.11	−0.61 (0.42)	0.00 0.98	−1.48 (63)	.15

## DISCUSSION

This study is one of the first of its kind to explore relationships between parents' voting behavior, political engagement beliefs, self‐efficacy, and parenting stress. Measures of political engagement beliefs correlated with one another, and likewise self‐efficacy measures correlated with one another. When looking at these constructs as grouped themes through factor analysis, contrary to our hypothesis there was no association between self‐efficacy and voting behavior, or between self‐efficacy and engagement beliefs. There was, however, a relationship between parents' political engagement beliefs and voting behavior.

### Parenting self‐efficacy, political engagement beliefs, and voting behavior

A relationship between political engagement beliefs and voting, yet no relationship between self‐efficacy and voting or between self‐efficacy and engagement can be interpreted in several ways. First, our survey may have failed to detect an actual relationship between parenting self‐efficacy and political activity, perhaps because the very brief survey failed to measure adequately the underlying constructs. The PTVQ piloted in the current study was built based on themes of LOC scales. Notably, validated LOC measures are typically longer in length (Halpert & Hill, 2011). Thus, this abbreviated 3‐question measure may not have adequately measured the constructs of self‐efficacy and LOC. While this study did not find a significant relationship, future studies of the potential relationship between parenting self‐efficacy and PE, using longer and better validated measures, might confirm the hypothesized relationship. Despite our negative findings, we think that the possibility that parents' PE may be a marker of parental empowerment bears further exploration. Parental empowerment is important to various aspects of child‐rearing and can be promoted in the health care setting through the parent‐provider relationship (Ashcraft et al., 2019). Thus, more research is needed in this area and clinicians are essential to this line of exploration.

It is also possible that there is no relationship between parenting self‐efficacy (personal empowerment) and political participation. There are several factors in the literature that influence political engagement, (“Who Votes, Who Doesn't, and Why,”, 2006) and it is possible that parenting self‐efficacy does not rank high in this list. Future studies could explore why some parents, who may not feel in charge in their parenting role, nonetheless commit to political engagement.

### Discussing voting with parents

This study explored parents' general openness to talking to their child's primary care physician about political participation, where over half of parents were open to having non‐partisan conversations, while nearly a quarter felt that doctors *should* ask about voting. The views of the AMA and AAP support physicians and health systems in encouraging patients to participate in elections (Chomilo et al., 2021; Why It's OK for Doctors to Ask Their Patients about Voting, 2021). It is important that physicians do their due diligence in discussing with patients about matters that affect patients' health and wellbeing. There are several factors of our patient's lives which are perceived as less comfortable for patients to discuss, yet still essential to ask about, such as sexual health and weight. Likewise, political engagement is important for our patients' health and wellness. It is possible that respondents in this study felt less comfortable discussing voting because they are not voting. Similarly, there can sometimes be discomfort in discussing sexual health among those with riskier sexual practices. Yet, in both examples, it is still critical to ask. Thus, the low numbers of participants in this study responding that doctors should ask about voting does not mean that doctors should not ask.

Beyond the clinical setting, physician engagement in community and advocacy issues is important in improving the health of patient populations served (Gruen, 2004). Physician advocacy has led to improvements in environmental issues such as lead, asthma, and social determinants of health. Likewise, voting is important to community well‐being (Liggett et al., 2014; Peeples, 2020). Advocacy for political participation has been successful in the clinical setting, and is supported by national physician organizations (Brown et al., 2020; Chomilo et al., 2021). Thus, physicians should feel empowered to discuss political participation with patients.

Importantly, within our convenience sample of parents seeking pediatric care for their children in an urban safety‐net hospital, the rate of political engagement was low: Only 37% of participants reported participation in the major 2020 election and 34% in the 2018 election. This is significantly lower than the general population, where 67% of eligible voters in the general US population voted in 2020, and 74% of eligible voters in Ohio voted in 2020 (Bureau, 2022). In Cuyahoga County, where the current study took place, 71% of eligible voters participated in the 2020 election, ranging from 10% to 77% of eligible voters participating within the city precincts (2020 Official Elections Results, 2022). The precinct in which this pediatric practice is located had a voting rate of 45.7% (2020 Official Elections Results, 2022). Thus, it is possible that this study may have yielded different findings in a community that is more politically active than this sample. Further work is needed to explore barriers to voting in this population, and ultimately encourage political participation among families.

### Limitations

There were several limitations to this pilot study. This study was conducted in a busy urban well‐child clinic and some parents were not interested in participating. The number of parents declining to participate was not tracked. However, generally, most parents were open to filling out the survey while awaiting care. Another limitation was the convenience sample. Participants were surveyed during times of researchers' availability to distribute the survey. Convenience sampling may not be fully representative of the general population, while more efficient and less costly (Jager et al., 2017). This pilot study took place in a metropolitan safety‐net hospital system, and the sample's responses may not be generalizable to other populations. Given the known relationship between SES and voting, survey responses may be different in other demographic populations compared to the study sample. Finally, as noted above, our exploratory survey instrument used a small number of questions to gauge the core concepts of political engagement, parenting self‐efficacy, and parenting stress; studies with longer, more robust surveys could come to different conclusions. Our survey was available only in English, thus excluding parents who were not comfortable using English.

### Future directions

Currently no validated tool exists to measure the relationship between parenting efficacy and political engagement. LOC is related to voting behavior, and likewise, parental self‐efficacy influences parenting outcomes (Freed & Tompson, 2011; Nowicki et al., 2017; Rosen & Salling, 1971). Further exploration is needed to determine a potential relationship between parental self‐efficacy and political engagement, including further testing and improvement of the research tool. The long‐term, societal impact of this line of research is that voting discussions between primary care doctors and parents could become normal parts of preventive health education, which could lead to greater empowerment among parents and a higher level of civic engagement within communities.

## CONFLICT OF INTEREST STATEMENT

The authors have no conflicts of interest to disclose.

## APPENDIX A SURVEY INSTRUMENT

                                                                              Today's Date __________

**Parents’ Thoughts about Voting Questionnaire (PTVQ)**

This survey will help us understand parents’ thoughts about voting.

For each statement, circle a number to show how strongly you agree or disagree with it.

Example: If you *agree* that “Voting is important in a democracy” you might circle

                                   disagree 1.2.3.4.5.6.7.8.9.10 agree

**Table:**

1	I feel I am in control of my own life.	disagree 1.2.3.4.5.6.7.8.9.10 agree
2	I feel I am in control of what happens to my child.	disagree 1.2.3.4.5.6.7.8.9.10 agree
3	I feel very stressed by my responsibilities as a parent.	disagree 1.2.3.4.5.6.7.8.9.10 agree
4	Voting is an important part of citizenship.	disagree 1.2.3.4.5.6.7.8.9.10 agree
5	Voting by parents can make a difference in their children's health.	disagree 1.2.3.4.5.6.7.8.9.10 agree
6	My vote matters.	disagree 1.2.3.4.5.6.7.8.9.10 agree
7	I would feel comfortable talking about voting with my child's doctor, as long as we didn't talk about the sides.	disagree 1.2.3.4.5.6.7.8.9.10 agree
8	Pediatricians and family doctors should ask parents about voting.	disagree 1.2.3.4.5.6.7.8.9.10 agree
9	If more parents voted, children would be better off.	disagree 1.2.3.4.5.6.7.8.9.10 agree
10	I want my child to grow up to be an engaged citizen.	disagree 1.2.3.4.5.6.7.8.9.10 agree

What year were you born in? _________ What month? __________

What is your main parenting role?

     mother   father   grandparent   foster parent   step parent   other__________

                                                        (More on back)

In what income group would you describe your family?

      Lower income         middle income         higher income         no answer

Do you consider yourself to be a “person of color” (vs. “white”)      YES   NO   no answer

Did you vote in the November 3, 2020 election?      YES      NO      Don't know

If not, why not? Please circle one or more:

       Couldn't make time Not registered not eligible other _______________________

Did you vote in the 2018 congressional election?      YES      NO      Don't know

If not, why not? Please circle one or more:

Couldn't make time Not registered not eligible other _______________________

*Thank you for taking time to do this survey! Please add comments below or on the back…*.
